# Effect of fenugreek seeds and *Bacillus*-based direct-fed microbials on the growth performance, blood biochemicals, and intestinal histomorphology of broiler chickens

**DOI:** 10.3389/fvets.2023.1298587

**Published:** 2023-11-28

**Authors:** Deependra Paneru, Guillermo Tellez-Isaias, Margarita A. Arreguin-Nava, Nicholas Romano, Walter G. Bottje, Emmanuel Asiamah, Ahmed A. A. Abdel-Wareth, Jayant Lohakare

**Affiliations:** ^1^Department of Poultry Science, University of Georgia, Athens, GA, United States; ^2^Department of Poultry Science, University of Arkansas, Fayetteville, AR, United States; ^3^Eco-Bio LLC, Fayetteville, AR, United States; ^4^Virginia Cooperative Extension, College of Agriculture, Virginia State University, Petersburg, VA, United States; ^5^Department of Agriculture, University of Arkansas at Pine Bluff, Pine Bluff, AR, United States; ^6^Department of Animal and Poultry Production, Faculty of Agriculture, South Valley University, Qena, Egypt; ^7^Poultry Center, Cooperative Agricultural Research Center, Prairie View A&M University, Prairie View, TX, United States

**Keywords:** broilers, nutrition, intestinal morphology, fenugreek, *Bacillus*-DFM

## Abstract

**Background:**

The objective of the present study was to evaluate the potential synergistic impact of the combination of fenugreek seeds (FS) and *Bacillus*-based direct-fed microbials (DFM) on growth performance, intestinal health, and hematological parameters of broiler chickens.

**Methods:**

A total of 160 one-day-old (Ross 308) broiler chicks were randomly assigned to a 2 × 2 factorial arrangement, with two levels of FS (0 and 5 g/kg) and two levels of *Bacillus*-DFM (0 and 0.1 g/kg), with five replicates of 8 birds each.

**Results:**

The result showed that dietary supplementation of FS at 5 g/kg did not improve the growth performance of broilers but impaired the early growth performance by reducing body weight gain and increasing feed conversion ratio, which was recovered during finisher phase. Dietary supplementation of *Bacillus*-based DFM at 0.1 g/kg did not affect the performance variables but increased the feed conversion ratio. The interaction of fenugreek seeds and *Bacillus*-based DFM showed synergistic effects on growth performance during the later stages of production. However, antagonistic effects were observed on the blood parameters and the gut morphology.

**Conclusion:**

This study demonstrated that FS and DFM had different effects on the broiler health and production depending on the phase of production. The interaction between FS and DFM revealed synergistic effects on growth performance during the finisher phase, but antagonistic effects on blood parameters and gut morphology. Further studies are needed to elucidate the underlying mechanisms and optimize the dosage and combination of FS and DFM for broiler health and production.

## Introduction

In recent years, there has been growing concern about the impact of antibiotics used in poultry feed on public health and the environment, prompting a need to reassess this controversial practice (1–4). The addition of subtherapeutic doses of antibiotics to poultry feed has resulted in the emergence of antibiotic-resistant pathogenic bacteria (5) and the accumulation of antibiotic residues in poultry meat and eggs (6). Consequently, the European Commission has imposed restrictions on the use of antibiotic growth promoters AGPs (7), and the United States Food and Drug Administration has requested that pharmaceutical companies cease labeling antimicrobials as growth promoters in agriculture animals under Guidance-213 and that antibiotic can only be administered for therapeutic purposes under veterinarian supervision (8). This has intensified the search for alternative, non-antibiotic solutions to maintain or improve poultry health and performance while addressing health and safety concerns for consumers, which drives the growing preference for natural and safe alternatives such as phytogenic and direct-fed microbials (DFM) (9, 10).

Fenugreek (*Trigonella foenum-graecum*) is a potential alternative to antibiotics in poultry due to its nutritional and bioactive properties. The high soluble fiber content of fenugreek seeds (FS) is thought to improve gut health by promoting the growth of beneficial bacteria and reducing harmful bacteria (11). This, in turn, may lead to improved feed efficiency and digestive health, as well as increased growth and disease resistance in poultry. Additionally, FS contains various bioactive compounds, such as saponins, alkaloids, and flavonoids, which possess health-promoting effects (12). These compounds may modulate the immune system, provide antioxidant activity, and regulate hormones, potentially contributing to improved health and performance in poultry (13–15). FS has anti-inflammatory and antimicrobial properties, which may help prevent and control infections in poultry (16).

*Bacillus*-based DFMs, another promising alternative to AGPs, are probiotics intended to improve gut health and enhance nutrient utilization in broiler chickens (17, 18). The mode of action of DFMs includes (a) augmentation of beneficial bacteria populations, such as *Lactobacillus* and *Bifidobacterium* spp., (b) reduction of pathogenic bacteria through competitive exclusion or by producing bacteriocins, (c) stimulation of metabolism by increasing both endogenous and exogenous digestive enzymes, (d) amelioration of ammonia production, (e) neutralization of enterotoxins (f) enhancement of the immune system, and (g) generation of beneficial metabolic byproducts, such as volatile fatty acids (19, 20).

Furthermore, as effective natural probiotics and phytogenic become more widely available, they are becoming a popular alternative to antibiotics in chickens to boost productivity and reproduction (16). Fenugreek seeds, which are high in dietary fiber, soluble fiber, and biologically active phytochemicals, act as a prebiotic (16) and may have synergistic effects, offering health-promoting benefits or stimulating broiler growth performance. However, few research have focused on the utilization of fenugreek seeds and *Bacillus*-DFMs to improve broiler chicken growth performance, gut integrity, and health. Furthermore, the synergistic benefits of probiotics and fenugreek seeds in broilers have not been consistently established in previous studies. It is hypothesized that fenugreek seeds and DFM will influence intestinal health and improve intestinal absorption, which altogether improves performance.

For this reason, the proposed studies aimed to assess the potential synergistic impact of the combination of FS and *Bacillus*-based DFM on growth performance, intestinal health, blood biochemical, and hematological parameters of broiler chickens.

## Materials and methods

All the experimental procedures were conducted in accordance with the ethical guidelines set by the Institutional Animal Care and Use Committee (IACUC) of the University of Arkansas at Pine Bluff and were approved under protocol number #UAPB2020-04. The protocols adhered to the principles outlined in the National Institutes of Health’s (NIH) Guide for the Care and Use of Laboratory Animals to ensure the humane treatment of all animals involved in the experiments.

### Preparation of fenugreek seeds and *Bacillus*-based direct-fed microbials

Fenugreek seeds (Deep Foods Inc., Union, NJ) were procured from a local supplier in Little Rock, AR. The seeds were ground using a Grinding Grain Mill (Thomas Scientific, Swedesboro, NJ) utilizing a 1 mm sieve to achieve a medium consistency. The ground seeds were then added on the top of starter and finisher diets at the rate of 5 g/kg of the diets in the experimental groups requiring FS. The GC–MS analysis of the fenugreek seed extract was done previously using gas chromatography–mass spectrometry (21). The extract of fenugreek seeds powder contained several active compounds, with the most abundant ones being 2-phenyl-4-(trimethylsilyl) furan at 25.45%, followed by 9,17-octadecadienal at 11.26%, curlone at 10.92%, α-curcumene at 10.31%, and cis,cis,cis-7,10,13-hexadecatrienal at 8.1%. Additionally, other compounds were present in smaller percentages, including tetradecanal, β-sesquiphellandrene, zingiberene, β-bisabolene, 7-methoxymethyl-2,7-dimethylcyclohepta-1,3,5-triene, caryophyllene oxide, 5-fluoro-1,1,3,3-tetramethyl-1,3-dihydroisobenzofuran, thymol, linoleic acid, p-cymene, α–tumerone, trans-caryophyllene, palmitic acid, and benzaldehyde, each in varying proportions.

Norum™, a patented *Bacillus*-direct fed microbial (DFM) from the University of Arkansas, consisting of three *Bacillus* strains selected for their enzyme activity. Norum™ was sourced from Eco-Bio/Euxxis Bioscience LLC and added to feed at 0.1 g/kg for experimental groups. The *Bacillus* strains within Norum™ were identified through whole-genome sequencing as *Bacillus subtilis* (AM1002), *Bacillus amyloliquefaciens* (AM0938), and *Bacillus licheniformis* (JD17) (22).

### Birds and experimental design

One-day-old male broiler chicks (Ross 308; *n* = 160) were obtained from a commercial hatchery (Keith Smith, Hot Springs, AR). Upon arrival, the chicks were weighed and randomly assigned to four treatment groups in a 2 × 2 factorial design, with five pens per treatment and 8 birds per pen. Each floor pen (180 × 90 × 48 cm^3^) was covered with pine wood shavings and equipped with separate feeders and drinkers (Harris Farms, Tractor Supply Co., Pine Bluff, AR). The housing temperature was carefully controlled throughout the growth cycle of the birds, starting at 33.5°C at the time of placement and gradually decreasing to 23.6°C by day 42 following the breeder recommendation. The birds were monitored twice daily to ensure their well-being, and factors such as room temperature, bird condition, mortality, and the availability of feed and water were checked during each inspection to ensure optimal conditions for growth and health. The treatments consisted of a corn-soybean meal basal diet (Table 1) supplemented with two levels of FS (F0 = 0 g/kg FS and F1 = 5 g/kg FS) and two levels of DFM (D0 = 0 g/kg DFM and D1 = 0.1 g/kg DFM). The diets were formulated to meet or exceed the nutrient requirements of Ross 308, with two-phase feeding system (starter, 0 to 21 days) and (finisher, 22 to 42 days) (23). Feed was provided in mash form. All birds were offered *ad libitum* access to feed and water throughout the study period.

**Table 1:** Ingredient composition of the basal diet for starter and finisher growth periods, as-fed basis.

Ingredient (%)	Starter (0 to 21 days)	Finisher (22 to 42 days)
Corn	59.4	66.5
Soybean meal	32.9	25.3
Pro plus^1^	2.5	0
Meat and bone meal, 50%	0	2.5
Poultry oil	2.01	3.14
Sodium chloride	0.38	0.31
Sodium bicarbonate	0	0.05
Limestone	0.8	0.7
Dicalcium phosphate	1.13	0.85
Vitamin Premix^2^	0.1	0.1
Mineral premix^3^	0.1	0.1
Choline Chloride	0.1	0.1
Selenium PMX 0.06%	0.02	0.02
Santoquin	0.02	0.02
L-Lysine HCL	0.17	0.10
DL-Methionine	0.3	0.21
L-Threonine	0.11	0.05
Copper chloride	0.02	0
Xylanase	0	0.01
Phytase	0.02	0.02
Total	100	100
Calculated analysis		
ME (kcal/kg)	3015.00	3090.00
Crude protein (%)	22.30	20.00
Lysine (%)	1.18	1.05
Methionine (%)	0.59	0.53
Total calcium (%)	0.90	0.84
Available phosphorus (%)	0.45	0.42

1 Pro-Plus is an animal by-product blend with a crude protein content of 60% (H. J. Baker & Bros. Inc., Little Rock, AR).

2 Vitamin premix (provided the following per kilogram of diet): vitamin A (trans retinyl acetate), 3,600 IU; vitamin D_3_ (cholecalciferol), 800 IU; vitamin E (DL-α-tocopheryl acetate), 7.2 mg; vitamin K3, 1.6 mg; thiamine, 0.72 mg; riboflavin, 3.3 mg; niacin, 0.4 mg; pyridoxin, 1.2 mg; cobalamine, 0.6 mg; folic acid, 0.5 mg; choline chloride, 200 mg.

3 Mineral premix (provided the following per kilogram of diet): Mn (from MnSO_4·_H_2_O), 40 mg; Zn (from ZnO), 40 mg; Fe (from FeSO_4·_7H_2_O), 20 mg; Cu (from CuSO_4·_5H^2^O), 4 mg; I [from Ca(IO_3_)_2·_H_2_O], 0.64 mg; Se (from sodium selenite), 0.08 mg.

### Proximate and mineral nutrient analysis

Feed samples were collected during bagging and analyzed for proximate and mineral nutrients at University of Arkansas (Fayetteville, AR). Table 2 shows the analyzed composition of all experimental diets. Dry matter was determined by drying the samples at 55°C overnight. Crude protein was determined by the Kjeldahl method as described by (24). Ether extract was determined by the Soxhlet method following the procedure of AOSC (25). Neutral detergent fiber and acid detergent fiber were determined by the methods of (26) and (27), respectively. Mineral nutrients were determined by digesting the samples with HNO_3_ and H_2_O_2_ and measuring them by atomic absorption spectrophotometry as described by (28).

**Table 2:** Analyzed composition of the experimental diets for starter and finisher periods, as DM basis.^1^

Item, %	Starter				Finisher			
	F0		F1		F0		F1	
	D0	D1	D0	D1	D0	D1	D0	D1
Dry matter	91.3	90.8	90.9	91	91.3	91	91	91.1
Crude protein	24.4	23.5	24.5	25.7	21.4	22.1	22.1	21.8
Ether extract	5.3	5.82	5.63	5.36	7.06	7.09	7.85	6.61
NDF	14.5	13.2	12.4	11.7	11.2	13.5	11.4	13.7
ADF	6	5.7	5.7	5.7	5.8	6.3	5.4	5.5

1 F0 = first level of fenugreek seeds, 0 g/kg of diet; F1 = second level of fenugreek seeds, 5 g/kg of diet; D0 = first level of *Bacillus*-based direct-fed microbial, 0 g/kg of diet; D1 = Second level of *Bacillus*-based direct-fed microbial, 0.1 g/kg of diet.

### Data collection

Data on live body weight (BW) and feed intake (FI) per pen were recorded at the end of both the starter period (d 21) and the finisher period (d 42) and used to calculate the mean values for body weight gain (BWG), FI, and feed conversion ratio (FCR). Mortality was recorded as it occurred.

At the end of the experimental period (d 42), two birds from each pen with a mean body weight were selected and humanely euthanized via rapid decapitation technique. Blood was collected from the jugular vein into 3 ml BD Vacutainer EDTA Blood Collection Tubes (Becton, Dickinson and Company, Franklin Lakes, NJ) for whole blood analysis. The whole blood samples were analyzed for white blood cell (WBC) count, hemoglobin, total protein, heterophil, lymphocyte, monocyte, and basophil counts. Another 3 ml of blood was collected in BD Vacutainer Serum Tubes (Becton, Dickinson and Company, Franklin Lakes, NJ) to obtain blood serum. The collected blood was allowed to clot for approximately 2 h at room temperature and then centrifuged at 2000 x g for 15 min using a Centrifuge 5430R (Eppendorf SE, Enfield, CT) to separate the serum. The serum samples were analyzed for cholesterol, albumin (AL), globulin (GL), albumin/globulin ratio (A: G), alanine aminotransferase (ALT), aspartate aminotransferase (AST), and gamma-glutamyl transferase (GGT). The whole blood and serum samples were analyzed by an external laboratory, the Arkansas State Veterinary Laboratory in Little Rock, AR.

The birds utilized for blood collection were then subjected to evisceration to obtain segments of the small intestine for detailed morphological examination. Samples of the mid-region of the jejunum and ileum were collected, washed with 10X phosphate-buffered saline, and then fixed in Bouin’s solution for 24 h. The samples were then preserved in 70% ethanol for 24 h and underwent a progressive dehydration process using increasing ethanol concentrations, followed by clearing in xylene. The samples were embedded in paraffin wax and sectioned at 5 μm thickness using a manual rotary microtome (Leica Biosystems, Buffalo Grove, IL). Two sections, taken at different depths, were stained with hematoxylin and eosin, and images were taken at 200x magnification. Five villi and crypts that were perpendicular to the muscularis mucosae and had a clear boundary with the adjacent structure were selected for further analysis. The height and depth of five randomly selected villi and crypts from each replicate were measured using Leica software (Leica DM3000, Leica Biosystems, Buffalo Grove, IL).

### Statistical analysis

Replicate pens were considered as the experimental unit for all analyses. The data were analyzed using a 2 × 2 factorial design with the Fit Model platform of JMP Pro 16.1 (SAS Institute Inc., Cary, NC) to examine the main effects and interactions between FS and Bacillus-DFM. Interaction effects that revealed significant differences were separated using the Tukey HSD, while main effects that displayed significant differences were separated using Student’s *t*-test. Statistical significance was set at a *p*-value of less than 0.05. The results were presented as least square means and the pooled standard error of the mean.

## Results

### Growth performance

The effects of fenugreek seeds (FS) and direct-fed microbials (DFM) on the growth performance of broiler chickens during the starter phase (0 to 21 days) are shown in Table 3. The main effect of FS was significant for body weight gain (BWG) and feed conversion ratio (FCR), but not for feed intake (FI). Chickens fed with F0 had higher BWG than those fed with F1 (*p* = 0.0027). Chickens fed with F0 also had lower FCR than those fed with F1 (*p* < 0.0001). There was no significant difference in FI between F0 and F1 (*p* = 0.2689). The main effect of DFM was not significant for any of the performance parameters. Chickens fed with D0 had similar BWG, FI and FCR as those fed with D1 (*p* > 0.05). There was no significant interaction between FS and DFM for any of the performance parameters (*p* > 0.05).

**Table 3:** Growth performance of birds during the starter phase (0–21 days).^1^

FS	DFM	Starter Phase (0 to 21 days)		
		BWG (g)	FI (g)	FCR (g)
Main effects				
F0		934^a^	1,210	1.30^b^
F1		863^b^	1,181	1.37^a^
	D0	918	1,214	1.32
	D1	879	1,176	1.34
SEM		14	18	0.01
*p*-values				
FS		0.0027	0.2689	<0.0001
DFM		0.0708	0.1627	0.1908
Interaction effects				
F0	D0	960	1,244	1.30
F0	D1	907	1,176	1.30
F1	D0	875	1,184	1.35
F1	D1	851	1,177	1.38
SEM		20	26	0.01
*p*-value				
FS × DFM		0.4707	0.2440	0.2347

^a,b^Means within a column not sharing a common superscript differ significantly (*p* < 0.05).

1 FS = fenugreek seeds; DFM = direct-fed microbials; F0 = 0 g/kg FS; F1 = 5 g/kg FS; D0 = 0 g/kg DFM; and D1 = 0.1 g/kg DFM.

The effects of fenugreek seeds (FS) and direct fed microbials (DFM) on the performance of broiler chickens during the finisher phase (22 to 42 days) are shown in Table 4. The main effects of FS and DFM were not significant for any of the performance parameters. Chickens fed with F0 had similar BWG, FI and FCR as those fed with F1 (*p* > 0.05). Chickens fed with D0 had similar BWG, FI and FCR as those fed with D1 (*p* > 0.05). There was a significant interaction between FS and DFM for BWG (*p* = 0.0353), but not for FI and FCR (*p* > 0.05). Chickens fed with F0D0 had the highest BWG among all the treatments, while chickens fed with F0D1 had the lowest BWG.

**Table 4:** Growth performance of birds during the finisher phase (22–42 days).^1^

FS	DFM	Finisher phase (22 to 42 days)		
		BWG (g)	FI (g)	FCR (g)
Main effects				
F0		1738	2,883	1.67
F1		1708	2,718	1.60
	D0	1762	2,747	1.57
	D1	1,683	2,854	1.71
SEM		49	66	0.05
*p*-values				
FS		0.6642	0.0972	0.3305
DFM		0.2683	0.2715	0.0706
Interaction effects				
F0	D0	1857^a^	2,852	1.55
F0	D1	1620^b^	2,914	1.80
F1	D0	1668^ab^	2,642	1.58
F1	D1	1747^ab^	2,794	1.62
SEM		69	94	0.07
*p*-value				
FS × DFM		0.0353	0.6428	0.1487

^a,b^Means within a column not sharing a common superscript differ significantly (*p* < 0.05).

1 FS = fenugreek seeds; DFM = direct-fed microbials; F0 = 0 g/kg FS; F1 = 5 g/kg FS; D0 = 0 g/kg DFM; and D1 = 0.1 g/kg DFM.

The effects of FS and DFM on the performance of broiler chickens during the overall phase (0 to 42 days) are shown in Table 5. The main effect of FS was not significant for BWG, FI or FCR. Chickens fed with F0 had similar BWG, FI and FCR as those fed with F1 (*p* > 0.05). The main effect of DFM was significant for FCR, but not for BWG and FI. Chickens fed with D0 had similar BWG and FI as those fed with D1 (*p* > 0.05). Chickens fed with D0 had lower FCR than those fed with D1 (*p* = 0.0495). There was a significant interaction between FS and DFM for BWG (*p* = 0.0328), but not for FI and FCR (*p* > 0.05). Chickens fed with F0D0 had the highest BWG among all the treatments, while chickens fed with F0D1 had the lowest BWG.

**Table 5:** Growth performance of birds during the overall phase (0–42 days).^1^

FS	DFM	Overall Phase (0–42 days)		
		BWG (g)	FI (g)	FCR (g)
Main effects				
F0		2,672	4,093	1.54
F1		2,570	3,898	1.52
	D0	2,680	3,961	1.48^b^
	D1	2,562	4,030	1.58^a^
SEM		52	73	0.03
*p*-values				
FS		0.1898	0.0786	0.6928
DFM		0.1317	0.5123	0.0495
Interaction effects				
F0	D0	2817^a^	4,096	1.46
F0	D1	2527^b^	4,090	1.62
F1	D0	2543^ab^	3,826	1.50
F1	D1	2598^ab^	3,971	1.54
SEM		74	104	0.05
*p*-value				
FS × DFM		0.0328	0.4777	0.1689

^a,b^Means within a column not sharing a common superscript differ significantly (*p* < 0.05).

1 FS = fenugreek seeds; DFM = direct-fed microbials; F0 = 0 g/kg FS; F1 = 5 g/kg FS; D0 = 0 g/kg DFM; and D1 = 0.1 g/kg DFM.

### Hematological parameters

The effects of fenugreek seeds (FS) and direct fed microbials (DFM) on the hematological parameters of broiler chickens on d 42 are shown in Table 6. The main effect DFM was significant for white blood cells (WBC), total protein (TP), lymphocyte count and heterophil: lymphocyte ratio (H: L), but not for heterophil count and basophil count. Chickens fed with F0 had similar WBC, TP, heterophil count, lymphocyte count, basophil count and H: L as those fed with F1 (*p* > 0.05). Chickens fed with F0 had lower Hb than those fed with F1 (*p* = 0.0268). Chickens fed with D0 had higher WBC and TP than those fed with D1 (*p* < 0.05). Chickens fed with D0 had similar Hb, heterophil count and basophil count as those fed with D1 (*p* > 0.05). Chickens fed with D0 had higher lymphocyte count and lower H: L than those fed with D1 (*p* < 0.05). There was a significant interaction between FS and DFM for WBC, TP, heterophil count and lymphocyte count (*p* < 0.05), but not for Hb, basophil count and H: L (*p* > 0.05). Chickens fed with F0D0 had the highest WBC, heterophil count and lymphocyte count among all the treatments, while chickens fed with F0D1 had the lowest WBC and lymphocyte count.

**Table 6:** Hematological parameters of birds fed fenugreek seeds (FS) and Direct fed microbials (DFM) on d 42.^1^

FS	DFM	WBC (10^3^/μl)	Hb (g/dl)	TP (g/dl)	HC (n/μl)	LC (*n*/μl)	BC (*n*/μl)	HLR (*n/n*)
Main effects								
F0		20.59	10.54^b^	2.82	8,879	5,935	5,696	1.76
F1		19.14	11.08^a^	2.84	8,601	5,526	4,957	1.80
	D0	21.34^a^	10.77	3.00^a^	8,720	6767^a^	5,815	1.34^b^
	D1	18.39^b^	10.86	2.67^b^	8,761	4694^b^	4,838	2.22^a^
SEM		0.96	0.27	0.08	424	551	427	0.17
*p*-values								
FS		0.2905	0.0268	0.8387	0.6469	0.6033	0.2300	0.8745
DFM		0.0368	0.7174	0.0053	0.9457	0.0120	0.1155	0.0009
Interaction effects								
F0	D0	24.00^a^	10.51	3.11	9839^a^	7803^a^	6,334	1.30
F0	D1	17.18^b^	10.57	2.53	7919^b^	4067^b^	5,057	2.23
F1	D0	18.67^ab^	11.03	2.89	7600^b^	5731^ab^	5,295	1.38
F1	D1	19.60^ab^	11.14	2.80	9603^a^	5321^ab^	4,619	2.23
SEM		1.50	0.27	0.13	571	885	686	0.23
*p*-values								
FS × DFM		0.0073	0.9134	0.0335	0.0025	0.0405	0.6221	0.8763

^a,b^Means within a column not sharing a common superscript differ significantly (*p* < 0.05).^1^FS = fenugreek seeds; DFM = direct-fed microbials; F0 = 0 g/kg FS; F1 = 5 g/kg FS; D0 = 0 g/kg DFM; and D1 = 0.1 g/kg DFM; WBC = white blood cell count; Hb = hemoglobin content; TP = total protein level; HC = heterophil count; LC = lymphocyte count; BC = basophil count; HLR = heterophil to lymphocyte ratio.

### Blood biochemical parameters

The effects of FS and DFM on the serum biochemical parameters of broiler chickens on d 42 are shown in Table 7. The main effects of FS and DFM were not significant for any of the serum biochemical parameters. Chickens fed with F0 had similar albumin, globulin, aspartate aminotransferase (AST), gamma-glutamyl transferase (GGT) and cholesterol as those fed with F1. Chickens fed with had similar albumin, globulin, AST, GGT and cholesterol as those fed with D1. There was no significant interaction between FS and DFM for any of the serum biochemical parameters (*p* > 0.05).

**Table 7:** Blood serum parameters of birds fed fenugreek seeds (FS) and Direct fed microbials (DFM) on d 42.^1^

FS	DFM	Albumin (g/dl)	Globulin (g/dl)	AST (IU/L)	GGT (IU/L)	Cholesterol (mg/dl)
Main effects						
F0		0.69	1.35	245	21.34	86.6
F1		0.69	1.33	252	22.40	89.6
	D0	0.70	1.34	243	21.39	88.0
	D1	0.68	1.34	253	22.35	88.2
SEM		0.03	0.07	15	0.88	4.9
*p*-values						
FS		0.9596	0.8114	0.7672	0.3991	0.6639
DFM		0.6132	0.9909	0.6354	0.4445	0.986
Interaction effects						
F0	D0	0.74	1.42	242	21.78	88.6
F0	D1	0.64	1.28	248	20.90	84.6
F1	D0	0.66	1.26	245	21.00	87.5
F1	D1	0.72	1.40	259	23.80	91.7
SEM		0.04	0.10	21	1.30	7.2
*p*-value						
FS × DFM		0.0674	0.1489	0.8678	0.1479	0.5581

1 FS = fenugreek seeds; DFM = direct-fed microbials; F0 = 0 g/kg FS; F1 = 5 g/kg FS; D0 = 0 g/kg DFM; and D1 = 0.1 g/kg DFM; AST = Aspartate aminotransferase; GGT = Gamma-glutamyl transferase.

### Intestinal histomorphology

**Figure 1:**
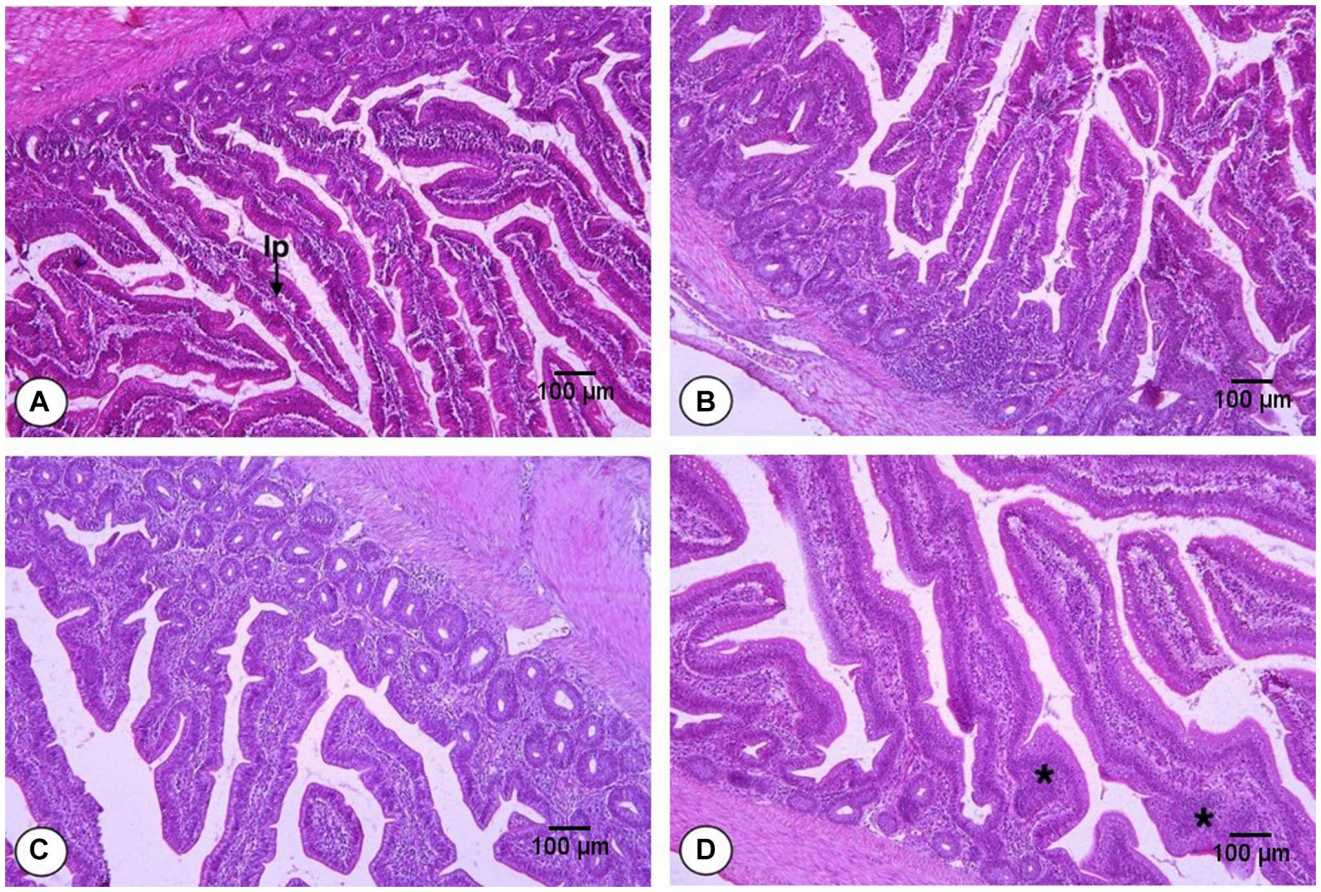
Histomorphology from the jejunum of broilers fed fenugreek seeds (FS) and Direct fed microbials (DFM) on d 42; F0D0 group. **(A)** F0D1 group, **(B)** F1D0 group, **(C)** F1D1 group (**D**; x200 magnification; H&E Staining). The “lp” represents the lamina propria, which is the smooth muscle fiber inside the intestinal villi. The asterisks (^*^) indicate the inflammation sites in the villi.

The effects of FS and DFM on the villus height (Vh), crypt depth (Cd) and villus height: crypt depth ratio (Vh:Cd) of the jejunum of broiler chickens at d 42 are shown in Table 8. The main effects of FS and DFM were significant for Cd and Vh:Cd, but not for Vh. Chickens fed with F0 had similar Vh as those fed with F1 (*p* > 0.05). Chickens fed with F0 had higher Cd and lower Vh:Cd than those fed with F1 (*p* < 0.05). Chickens fed with D0 had similar Vh as those fed with (*p* > 0.05). Chickens fed with D0 had lower Cd (*p* < 0.05) and numerically higher Vh:Cd (*p* = 0.0702) than those fed with D1. There was a significant interaction between FS and DFM for Vh and Cd (*p* < 0.05), but not for Vh:Cd (*p* > 0.05). Chickens fed with F1D0 had the highest Vh among all the treatments, while chickens fed with F0D1 had the highest Cd. However, FS also induced inflammation and monocyte infiltration in the jejunum epithelium and lamina propria (Figure 1), which may impair the gut barrier function and increase the susceptibility to pathogens.

**Table 8:** Jejunal morphology of birds fed fenugreek seeds (FS) and direct fed microbials (DFM) on d 42.^1^

FS	DFM	Vh (μm)	Cd (μm)	Vh:Cd
Main effects				
F0		1750	169^a^	10.51^b^
F1		1837	147^b^	12.55^a^
	D0	1764	149^b^	11.97
	D1	1823	168^a^	11.09
SEM		31	3	0.33
*p*-values				
FS		0.0557	<0.0001	0.0001
DFM		0.1878	<0.0001	0.0702
Interaction effects				
F0	D0	1674^b^	149^b^	11.33
F0	D1	1826^ab^	189^a^	9.70
F1	D0	1854^a^	148^b^	12.61
F1	D1	1820^ab^	146^b^	12.49
SEM		44	4	0.47
*p*-value				
FS × DFM		0.0415	<0.0001	0.1151

^a,b^Means within a column not sharing a common superscript differ significantly (*p* < 0.05).

1 FS = fenugreek seeds; DFM = direct-fed microbials; F0 = 0 g/kg FS; F1 = 5 g/kg FS; D0 = 0 g/kg DFM; and D1 = 0.1 g/kg DFM; Vh = villus height; Cd = crypt depth; Vh: Cd = villus height to crypt depth ratio.

**Figure 2:**
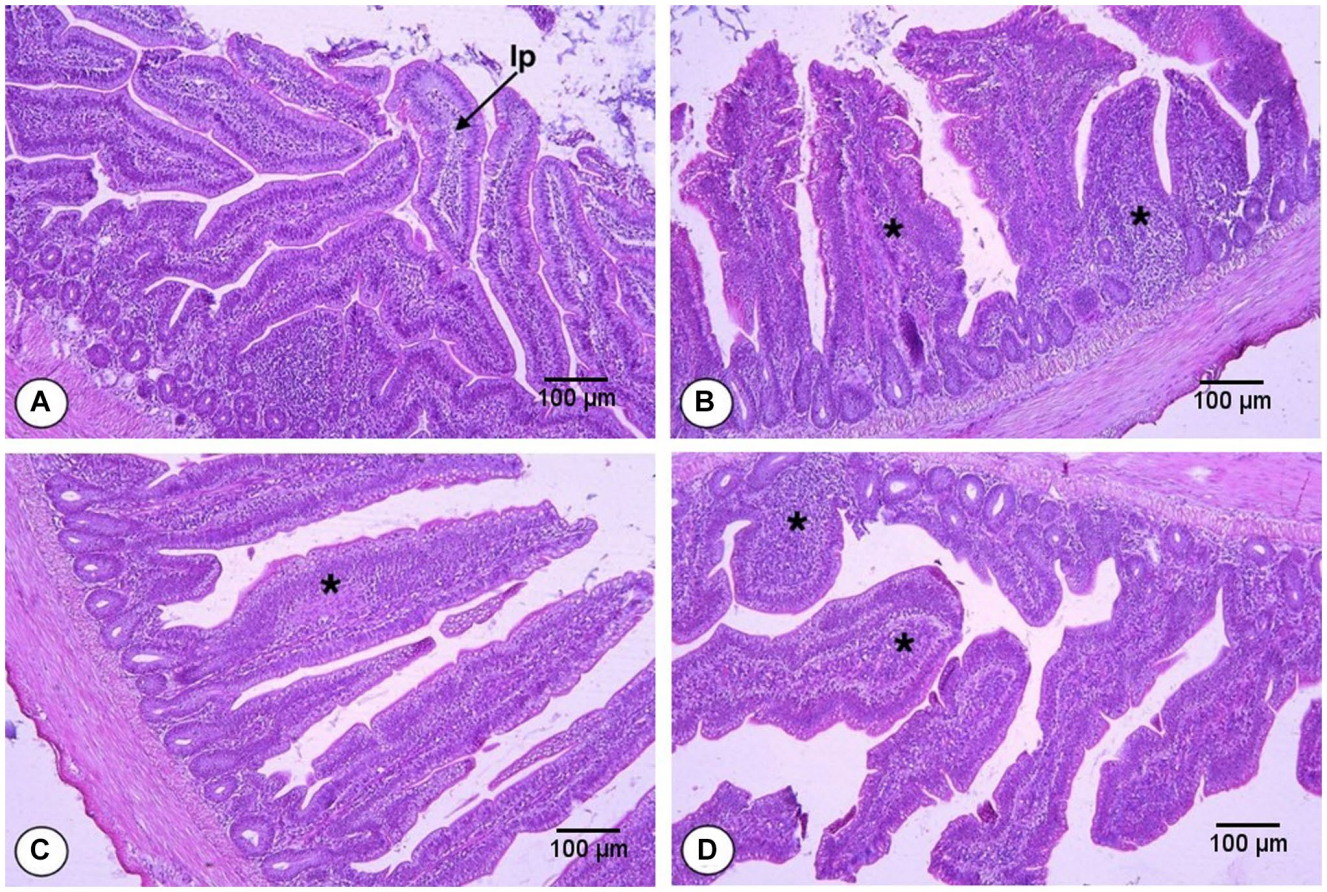
Histomorphology from the ileum of broilers fed fenugreek seeds (FS) and Direct fed microbials (DFM) on d 42; F0D0 group. **(A)** F0D1 group, **(B)** F1D0 group, **(C)** F1D1 group (**D**; x200 magnification; H&E Staining). The “lp” represents the lamina propria, which is the smooth muscle fiber inside the intestinal villi. The asterisks (^*^) indicate the inflammation sites in the villi.

The effects of FS and DFM on the Vh, Cd, and Vh:Cd of the ileum of broiler chickens at d 42 are shown in Table 9 and presented in Figure 2. The main effects of FS and DFM were significant for all the ileal parameters. Chickens fed with F0 had higher Vh, lower Cd and higher Vh:Cd than those fed with F1 (*p* < 0.05). Chickens fed with D0 had higher Vh, lower Cd and higher Vh:Cd than those fed with D1 (*p* < 0.05). There was a significant interaction between FS and DFM for Vh and Vh:Cd (*p* < 0.05), but not for Cd (*p* > 0.05). Chickens fed with F0D0 had the highest Vh and Vh:Cd among all the treatments, while chickens fed with F1D1 had the lowest Vh and Vh:Cd.

**Table 9:** Ileal morphology of birds fed fenugreek seeds (FS) and direct fed microbials (DFM) on d 42.^1^

FS	DFM	Vh (μm)	Cd (μm)	Vh: Cd
Main effects				
F0		742^a^	105^b^	7.24^a^
F1		704^b^	120^a^	5.94^b^
	D0	751^a^	107^b^	7.20^a^
	D1	695^b^	117^a^	5.98^b^
SEM		6	3	0.17
*p*-values				
FS		<0.0001	0.0003	<0.0001
DFM		<0.0001	0.0051	<0.0001
Interaction effects				
F0	D0	801^a^	98^b^	8.24^a^
F0	D1	683^b^	111^ab^	6.24^b^
F1	D0	701^b^	115^a^	6.15^b^
F1	D1	706^b^	124^a^	5.72^b^
SEM		8	4	0.24
*p*-value				
FS × DFM		<0.0001	0.6946	0.0021

^a,b^Means within a column not sharing a common superscript differ significantly (*p* < 0.05).

1 FS = fenugreek seeds; DFM = direct-fed microbials; F0 = 0 g/kg FS; F1 = 5 g/kg FS; D0 = 0 g/kg DFM; and D1 = 0.1 g/kg DFM; Vh = villus height; Cd = crypt depth; Vh: Cd = villus height to crypt depth ratio.

## Discussion

The present study investigated the effects of fenugreek seeds (FS) and direct-fed microbials (DFM) on the growth performance, blood parameters and gut health of broiler chickens. FS are seeds of the plant *Trigonella foenum-graecum* L., which are widely used as a spice and flavor enhancer in human food (29). The Food and Drug Administration has classified them as generally recognized as safe (GRAS), meaning that they have a history of safe use in food and are unlikely to cause adverse effects when consumed in normal amounts (30). However, some studies in animals have raised concerns about the potential toxicity of high doses of FS or their extracts, which contain high levels of flavonoid glycosides (31). Flavonoid glycosides are natural compounds that have antioxidant and anti-inflammatory properties, but they can also induce oxidative stress and organ damage when ingested in excess. The *Bacillus*-based DFM used in this study, Norum™ (Eco Bio/Euxxis Bioscience LLC, Fayetteville, AR), is a spore-based DFM culture consisting of three *Bacillus* strains: *Bacillus subtilis*, *B. amyloliquefaciens*, and *B. licheniformis* with a stable *Bacillus* spore content of ~3 × 10^11^ spores/g (22). These *Bacillus* strains can produce various enzymes (xylanase, cellulase, phytase, lipase, protease, and β-glucanase) that can improve the digestibility and nutrient availability of the feed, as well as modulate the intestinal microbiota and enhance the gut health and immunity of poultry (31, 32).

In the present study, the results showed that FS had a negative effect on the growth performance of broilers during the starter phase, but not during the finisher phase. DFM had a non-significant effect on the growth performance of broilers during either phase. There was an interaction between FS and DFM for BWG during the finisher and overall phases, indicating that the combination of FS and DFM may have some synergistic effects on broiler growth. The negative effect of FS on the growth performance of broilers during the starter phase may be attributed to the high saponin content in FS, which are known to have anti-nutritional properties such as reducing nutrient digestibility, interfering with bile acid absorption, and inhibiting intestinal enzymes (33). Earlier studies have demonstrated that broilers have little endogenous enzyme production during starter phase which might be negatively affected by the presence of antinutritional factors in the diet thus negatively affecting the growth performance during starter phase (34). FS may also have altered the intestinal microbiota of broilers, which play an important role in nutrient metabolism, immune modulation, and pathogen exclusion (35). These factors may have impaired the growth potential of broilers during the starter phase, when they are more sensitive to dietary changes and environmental stressors. The lack of effect of FS on the growth performance of broilers during the finisher phase may be due to their adaptation to the presence of FS in their diet over time and developed mechanisms to overcome its anti-nutritional effects. Broilers may also have benefited from some positive effects of FS on their health and immunity, which may have compensated for its negative effects on their growth. For instance, FS has been shown to have antioxidant, anti-inflammatory, antimicrobial, and immunomodulatory properties in various animal models (36, 37). It is also possible that broilers may have received adequate levels of nutrients from other sources in their diet, which may have mitigated the impact of FS on their nutrient utilization. It is worth noting that we observed similar results in our previous experiment with the inclusion of 2.5 to 10 g/kg of FS in the broiler diet (38).

The lack of effect of DFM on the growth performance of broilers during either phase may be due to several factors. DFM may not have been able to colonize or persist in the gastrointestinal tract of broilers, due to competition from native microorganisms or unfavorable environmental conditions. It is also possible that DFM may not have produced sufficient amounts or types of metabolites that could enhance the growth performance of broilers, such as short-chain fatty acids, vitamins, enzymes, or antimicrobial substances (39). DFM may not have interacted with the host immune system or intestinal epithelium in a way that could improve the growth performance of broilers, such as modulating inflammatory responses, enhancing barrier function, or stimulating mucosal immunity (38). The interaction between FS and DFM for BWG during the finisher and overall phases may be due to some synergistic or antagonistic effects between these two feed additives on broiler growth. For instance, FS and DFM may have synergized to improve the intestinal health and immunity of broilers by reducing oxidative stress, enhancing intestinal morphology, increasing beneficial bacteria, and suppressing pathogenic bacteria (40). Alternatively, FS and DFM may have antagonized each other by interfering with their respective modes of action or bioavailability. For example, FS may have reduced the efficacy or survival of DFM by inhibiting their growth or activity with its saponins or other phytochemicals. Conversely, DFM may have reduced the bioavailability or absorption of FS by degrading its active components or binding to them with their cell wall components.

The results showed that DFM had a significant effect on some hematological parameters, such as white blood cells (WBC), total protein (TP), lymphocyte count and heterophil: lymphocyte ratio (H: L), but not on others, such as hemoglobin (Hb), heterophil count and basophil count. FS had a significant effect only on Hb, but not on any other hematological parameters. There was an interaction between FS and DFM for WBC, TP, heterophil count and lymphocyte count, indicating that the combination of FS and DFM may have some synergistic or antagonistic effects on the blood profile of broilers. The reduction in WBC, TP, and lymphocyte count in broilers fed a DFM diet may be due to an alteration of the gut microbiome, leading to a reduction in systemic inflammation and a decrease in the production of cytokines, which are signaling molecules involved in the regulation of the immune system. An increase in the H:L ratio can indicate an activation of the immune system, as heterophils play a role in the body’s initial response to infection and inflammation (41). In such cases, the increase in heterophils can reflect an increase in the body’s ability to respond to and combat pathogens. It is not entirely clear why Bacillus-DFM specifically would increase the HLR, but some studies suggest that certain Bacillus strains have immune-stimulatory effects that can enhance the body’s ability to fight off infections and improve overall immunity (42). However, more research is needed to fully understand the mechanisms by which *Bacillus*-DFM might influence the H:L ratio and the immune system. The interaction of FS and DFM decreased the TP levels, WBC count, heterophil count, and lymphocyte count. This suggests that FS and DFM may have antagonistic effects on the blood parameters of broiler chickens. FS may contain some anti-nutritional factors, such as saponins or flavonoids, that may bind to or degrade some components of DFM, such as bacterial cell wall or enzymes, reducing their bioavailability or activity in the gut (43, 44). Alternatively, DFM may produce some metabolites or enzymes that may alter the pH or enzymatic activity in the gut, affecting the digestion or absorption of some components of FS, such as iron or flavonoids (45). These interactions may result in reduced efficacy or adverse effects of FS or DFM on the observed hematological parameters. The lack of effect of FS and DFM on the serum biochemical parameters may be attributed to several factors. FS and DFM may not have reached sufficient levels or durations in the gastrointestinal tract or bloodstream of broilers to exert their effects on the serum biochemical parameters. FS and DFM may not have altered the metabolic pathways or functions of the liver, kidney, or other organs that are involved in the synthesis or degradation of the serum biochemical parameters. FS and DFM may not have affected the homeostatic mechanisms that regulate the serum biochemical parameters within a narrow range. The serum biochemical parameters are important indicators of the health and physiological status of broilers, as they reflect the functions of various organs and systems (46). The normal ranges of these parameters may vary depending on the age, sex, breed, diet, environment, and health condition of broilers (47). The values of these parameters in this study were within the normal ranges reported for broiler chickens. This suggests that FS and DFM did not cause any adverse effects on the health and physiology of broilers.

The effect of FS on the intestinal morphology may be attributed to several factors. FS may contain some bioactive compounds, such as flavonoids or oligosaccharides, that may stimulate the growth and differentiation of intestinal cells, increasing the intestinal surface area and nutrient absorption capacity. Some studies have reported that FS supplementation can increase Vh, Cd, and Vh: Cd ratio of jejunum in broilers (48). However, another possible reason for the negative effect of FS on inflammation and monocyte infiltration in jejunum may be due to anti-nutritional factors, such as saponins, that may have cytotoxic and immunomodulatory effects on intestinal cells, inducing oxidative stress and inflammatory response (49). Some studies have reported that FS supplementation can cause inflammation and monocyte infiltration in jejunum in rats (50). Therefore, FS supplementation may have both positive and negative impacts on the jejunum morphology and gut health of broilers, which might depend on the dosage and growth stage of the birds.

One possible reason for the antagonistic effect of FS and DFM on Vh and Cd of jejunum is that FS and DFM may interfere with each other’s absorption or metabolism in the gut. FS may contain anti-nutritional factors, such as saponins or flavonoids, that may bind to or degrade the components of DFM, such as bacterial cell wall or enzymes, reducing their bioavailability or activity in the gut. Alternatively, DFM may produce some metabolites or enzymes that may alter the pH or enzymatic activity in the gut, affecting the digestion or absorption of some components of FS, such as iron or flavonoids. These interactions may result in reduced efficacy or adverse effects of FS or DFM on jejunum morphology and gut health. These results are consistent with our previous study that reported similar effects of FS on ileum morphology in broilers (38). Moreover, FS also induced inflammation and monocyte infiltration in the ileum epithelium and lamina propria (Figure 2), which may impair the gut barrier function and increase the susceptibility to pathogens. FS contains saponins, alkaloids, and flavonoids that can induce inflammation, which can negatively impact gut health and result in decreased ileum villus height to crypt depth ratio (50, 51). DFM supplementation decreased the Vh, increased the Cd and decreased the Vh:Cd of ileum in broilers, which is contrary to some previous studies that reported that DFM increased or did not affect the Vh and decreased the Cd of ileum in broilers (52, 53). However, the effects of DFM on intestinal morphology may depend on the strain, dose, form, duration, and combination of DFM used. The combination of FS and DFM decreased the Vh and Vh:Cd of ileum in broilers, suggesting the possible antagonistic effects on the ileum morphology of broilers. It is possible that FS may interfere with the colonization or activity of DFM in the intestine and reduce their beneficial effects on intestinal morphology. The interaction between FS and DFM for some intestinal parameters may be due to some synergistic or antagonistic effects between these two feed additives on the intestinal morphology and absorptive surface area of broilers. For instance, FS and DFM may have synergized to increase the Vh and Vh:Cd of broilers by enhancing their nutrient absorption and digestion with their enzymatic or prebiotic properties. Alternatively, FS and DFM may have antagonized each other by interfering with their respective modes of action or bioavailability. For example, FS may have reduced the efficacy or survival of DFM by inhibiting their growth or activity with its saponins or other phytochemicals. Conversely, DFM may have reduced the bioavailability or absorption of FS by degrading its active components or binding to them with their cell wall components.

## Conclusion

This study demonstrated that FS and DFM had different effects on the broiler health and production depending on the phase of production. The interaction between FS and DFM revealed synergistic effects on growth performance during the finisher phase, but antagonistic effects on blood parameters and gut morphology. Further studies are needed to elucidate the underlying mechanisms and optimize the dosage and combination of FS and DFM for broiler health and production. Future studies should also evaluate the effects of FS and DFM on other aspects such as immunity, microbiota, and carcass quality.

## Data availability statement

The raw data supporting the conclusions of this article will be made available by the authors, without undue reservation.

## Ethics statement

The animal study was approved by Institutional Animal Care and Use Committee (IACUC) of the University of Arkansas at Pine Bluff and were approved under protocol number #UAPB2020-04. The study was conducted in accordance with the local legislation and institutional requirements.

## Author contributions

DP: Data curation, Formal Analysis, Investigation, Methodology, Project administration, Supervision, Writing – original draft, Writing – review & editing. GT-I: Conceptualization, Validation, Writing – review & editing. MA-N: Conceptualization, Resources, Validation, Writing – review & editing. NR: Data curation, Formal Analysis, Investigation, Methodology, Writing – review & editing. WB: Conceptualization, Validation, Writing – review & editing. EA: Data curation, Formal Analysis, Methodology, Writing – review & editing. AA-W: Validation, Writing – review & editing. JL: Conceptualization, Data curation, Formal Analysis, Funding acquisition, Investigation, Methodology, Project administration, Validation, Writing – original draft, Writing – review & editing.
